# Evidence of membranolytic targeting and intracellular citrullination in neutrophils isolated from patients with rheumatoid arthritis

**DOI:** 10.1038/s41598-024-66516-w

**Published:** 2024-07-05

**Authors:** Fatemeh Moadab, Xiaoxing Wang, Ethan Le, Tal Gazitt, Rayan Najjar, J. Lee Nelson, Vijay Joshua, Vivianne Malmström, Keith Elkon, Caroline Grönwall, Tomas Mustelin

**Affiliations:** 1 Division of Rheumatology, Department of Medicine, University of Washington, 750 Republican Street, Room E507, Seattle, WA 99108 USA; 2 Division of Rheumatology, Department of Medicine Solna, Karolinska Institutet, Karolinska University Hospital, Stockholm, Sweden

**Keywords:** Immunology, Rheumatology

## Abstract

Anti-citrullinated protein autoantibodies (ACPA) are diagnostic for rheumatoid arthritis (RA). The antigens recognized by these autoantibodies are produced by protein arginine deiminases (PADs), particularly PAD4. However, it remains unknown why and how PAD4 causes this aberrant citrullination in RA. Here, we report that poly-perforin pores are present on freshly isolated neutrophils from RA patients, but not on healthy donor neutrophils. Neutrophils with perforin pores also contained intracellular citrullinated proteins in the region adjacent to the pores. This response was replicated in vitro by treating neutrophils with purified perforin, which generated intense dots of anti-perforin immunofluorescence, calcium influx, and intracellular citrullination. Extensive neutrophil killing in Felty’s syndrome, an aggressive form of RA, correlated with particularly high ACPA, and PAD4 autoantibodies. In contrast, other forms of death, including NETosis, apoptosis, and pyroptosis, produced minimal citrullination. We conclude that neutrophil targeting by perforin leading to intracellular citrullination takes place in patients with RA.

## Introduction

Rheumatoid arthritis (RA) is a systemic autoimmune disease, in which synovial joints are attacked by the innate and adaptive immune system^1^. Despite the recent increase in treatment options^2–4^, including several biologic and small-molecule drugs, many RA patients continue to suffer from insufficiently controlled disease.

By definition, all patients with classical ‘seropositive’ RA have circulating anti-citrullinated protein antibodies (ACPA), also referred to by the test used for detecting them, the anti-cyclic citrullinated peptide (anti-CCP) test. The specificity of ACPA for RA, together with an association of RA with single-nucleotide polymorphisms in *PADI4*^5,6^ and *PADI2*^7^, which encode enzymes with protein arginine deiminase (= citrullination) activity, suggest that protein citrullination plays an important and unique role in RA^8^. Based on the assumption that the immune system is tolerant of physiologically citrullinated proteins, we and others posit that aberrantly citrullinated proteins in RA patients stoke autoimmunity against such modified self^9^. It has been shown that citrullination can also alter the proteolytic processing of proteins and result in the presentation of unmodified peptides that the T cells have not seen before. Although the hypothesis that citrullination underpins RA remains to be critically tested in clinical trials, it is compatible with all existing insights into RA pathogenesis.

A key open question is why and how pathological citrullination occurs in RA patients. The first proposed mechanism^10^ was that citrullination accompanies neutrophil extrusion of decondensed nuclear DNA, known as neutrophil extracellular traps (NETs), which reportedly contain PADs and citrullinated proteins. However, NETs are released in a multitude of disease states, including bacterial infections^11^, SARS CoV2 infection^12^, acute respiratory distress syndrome^12,13^, and systemic lupus erythematosus^14,15^, yet none of these conditions are accompanied by ACPA, even in the context of a pre-disposing HLA haplotype. A second mechanism was published by Romero and co-workers^16^, who showed that Ca^2+^-influx into neutrophils triggers an extensive intracellular citrullination reaction, referred to as the ‘leukotoxic hypercitrullination’ response^16,17^. In this response, plasma membrane pores formed by polymerized perforin, or the membrane attack complex of complement, allow for sufficient quantities of Ca^2+^ to enter the cell to activate the intracellular PADs to citrullinate numerous intracellular proteins. It is currently not known if this mechanism operates in RA patients and, if so, whether it is relevant for the formation of ACPA and the development of RA.

While it remains unclear which cell lineage(s) is(are) responsible for the excessive citrullination, the neutrophil has emerged as a plausible candidate. Neutrophils accumulate in the synovial fluid in early stages of RA^18^. They express the highest levels of PAD4^8,16,17,19^, as well as PAD2^17,19^, and expose a portion of these enzymes on their surface^19^. A characteristic feature of PADs is their dependence on high calcium concentrations, with maximal activity seen at 5 to10 mM^17,20^, which is five orders of magnitude higher than its intracellular concentration ([Ca^2+^]_i_) in living cells, but only slightly higher than in extracellular fluid. Hence, it is not surprising that membrane-disrupting agents, such as polymerized perforin, can trigger PAD activation to rapidly citrullinate numerous intracellular proteins in neutrophils. Importantly, many of the citrullinated neutrophil proteins are also present in RA synovial fluid.

## Results

### Generation and validation of the ‘anti-knob’ perforin mAb

**Figure 1:**
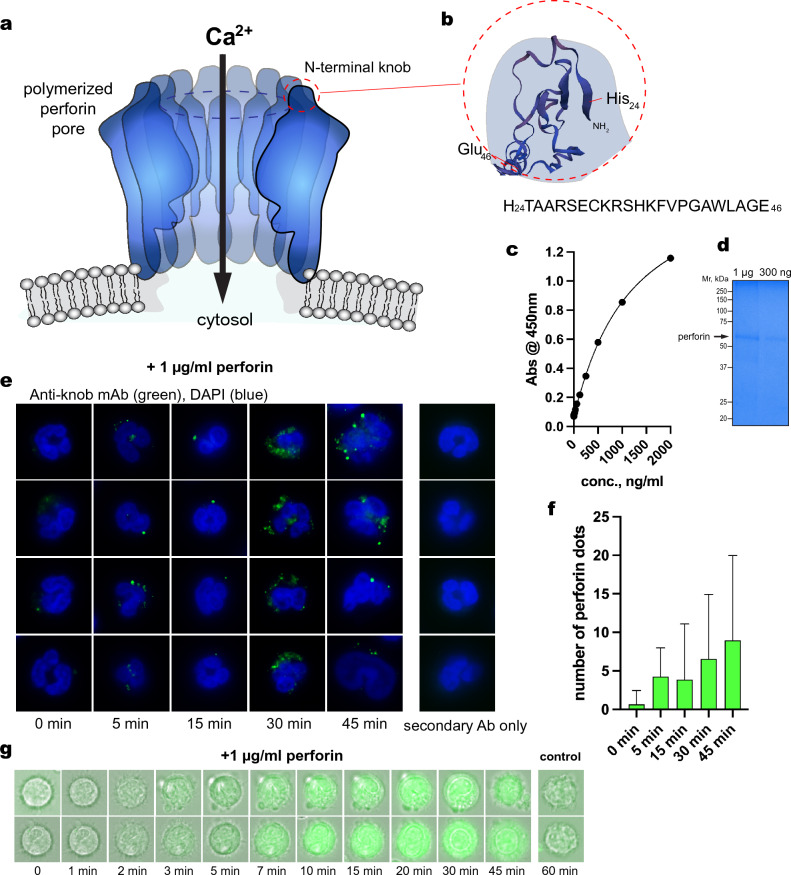
Generation and validation of the knob mAb (anti-perforinN). (**a**) The 3-dimensional structure of the poly-perforin pore. (**b**) Close up of the N-terminal knob highlighting the peptide His_24_–Glu_46_, which was used as immunogen conjugated to keyhole limpet hemocyanin. (**c**) ELISA of anti-knob mAb 4G10 using purified perforin. (**d**) Coomassie Blue stain of 1 µg and 0.3 µg of the purified perforin used in panel c. (**e**) Immunofluorescence staining with anti-knob mAb 4G10 of neutrophils treated with 1 µg/ml of purified perforin for the indicated times. (**f**) Quantitation of the response in panel e expressed as the number of brightly stained dots per neutrophil. (**g**) Live cell imaging of two neutrophils loaded with the Fluo-5N calcium indicator and treated with 1 µg/ml of perforin at 37 °C.

To be able to detect polymerized perforin pores on the surface of freshly isolated RA neutrophils, we developed a monoclonal antibody against the exposed N-terminal knob of perforin (Fig. 1a,b). This anti-knob mAb (clone 4G10) was validated by ELISA against purified human perforin (Fig. 1c,d) and immunofluorescence staining of neutrophils treated over a time-course with 1 µg/ml purified (monomeric) perforin, which resulted in a time-dependent appearance of brightly stained dots on the cells (Fig. 1e,f).

Pores formed by perforin polymerization in the plasma membrane of target cells mediate a rapid influx of Ca^2+^ down its 10,000-fold concentration gradient from the extracellular 2.2–2.6 mM to intracellularly around 150 nM. To demonstrate that such pores were indeed visualized by our mAb, live neutrophils were loaded with the calcium indicator Fluo-5N and then treated with 1 µg/ml perforin. As shown in Fig. 1g, the intracellular fluorescence began to rise after a few minutes, reaching a plateau around 15–20 min. By mass spectrometry, the treated neutrophils contained numerous citrullinated proteins (Supplemental Table S1). Taken together, these data leave no doubt that our knob mAb can visualize poly-perforin pores.

### Detection of perforin pores in RA patient neutrophils

**Figure 2:**
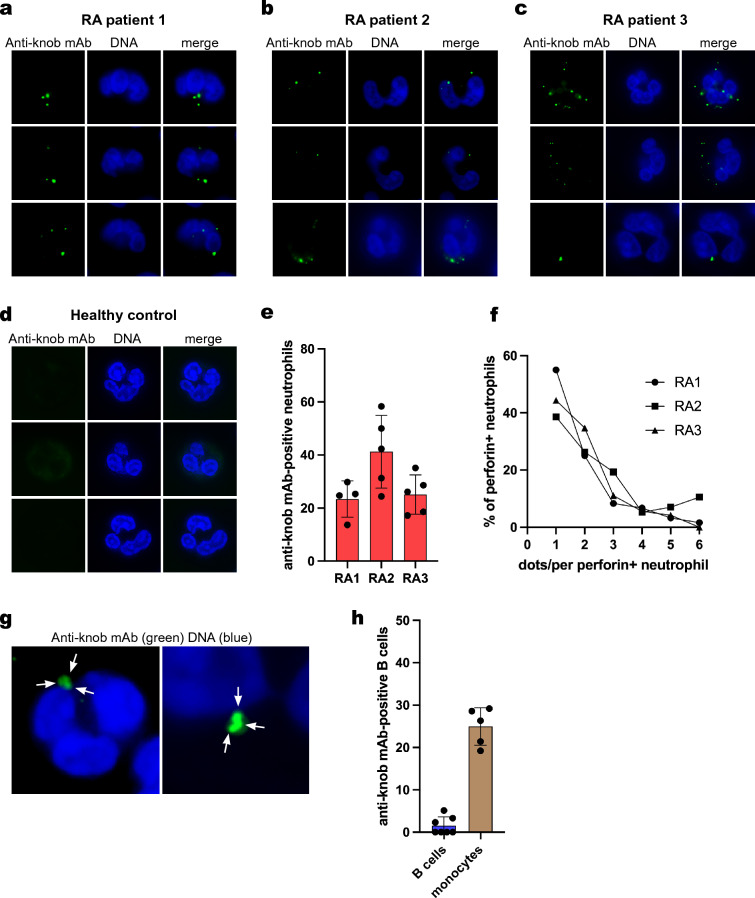
Poly-perforin pores on freshly isolated untreated RA neutrophils. (**a**) Three representative neutrophils from an RA patient stained with the anti-knob mAb. (**b**) Three representative neutrophils from a second RA patient. (**c**) Three representative neutrophils from a third RA patient. (**d**) Similar staining of three representative neutrophils from a healthy donor. (**e**) Quantitation of the perforin pore-positive neutrophils in the same three patients, expressed as percent of all neutrophils, in four different fields containing approximately 100 cells. (**f**) Quantitation of the number of bright perforin dots on each of the perforin-positive neutrophils from the same three RA patients. (**g**) Close ups of two clusters of 3–4 bright dots. (**h**) quantitation of perforin pores in RA B cells and monocytes.

When untreated freshly isolated neutrophils from RA patients were stained with the anti-knob mAb, it was evident that small intensely staining dots were present in a number of neutrophils (Fig. 2a–c). They were similar in size and intensity to the ones seen after adding perforin in Fig. 1, but even more uniform in size. In contrast, neutrophils from healthy donors did not have any perforin pores (Fig. 2d). The three illustrated RA patients had different percentages of positive neutrophils (Fig. 2e); the first patient (RA1) had fewer and also had a low disease activity score (CDAI = 0), while RA2 had the most active disease (CDAI = 38) and 43% perforin pore positive neutrophils. Nevertheless, in each positive cell, the numbers of brightly stained dots were similar between the 3 patients (Fig. 2f); except that neutrophils from the second patient had somewhat more dots on average, including a few with 6 or more dots. While most dots were of a relatively uniform size, some were larger and/or irregular in shape, perhaps representing several closely spaced pores. Figure 2g shows two examples of such clusters. In contrast to neutrophils, only occasional B lymphocytes from RA patients had perforin pores (Fig. 2h), while approximately 20% of monocytes had 1–5 perforin pores, most of them only one (Fig. 2h and Supplemental Fig. S1). We conclude that a portion of circulating neutrophils, and to a lesser extent monocytes, freshly isolated from RA patients have poly-perforin pores on their surface. Even if some patient-to-patient variations were observed in percent positive neutrophils and in numbers of pores per cell, every one of the 12 RA patients examined had neutrophils with poly-perforin pores.

### Citrullination in perforin-positive RA neutrophils

Citrullination is technically challenging to quantitate. We tested a panel of RA patient-derived monoclonal ACPA generated at the Karolinska Institutet and found that one of them, L201_10D07^21^, was particularly suitable for our purpose: it did not stain neutrophils from healthy donors, but stained them in a granular pattern after a brief treatment with 2 µM ionomycin, which induces a strong Ca^2+^-dependent intracellular citrullination of numerous proteins^16,17^ (Supplemental Fig. S2). The L201_10D07 mAb also stained most RA neutrophils very faintly, but approximately 2% of them more intensely (Supplemental Fig. S3). Another ACPA mAb (1235_04C03) also showed this pattern. Neutrophils from healthy donors were negative.

**Figure 3:**
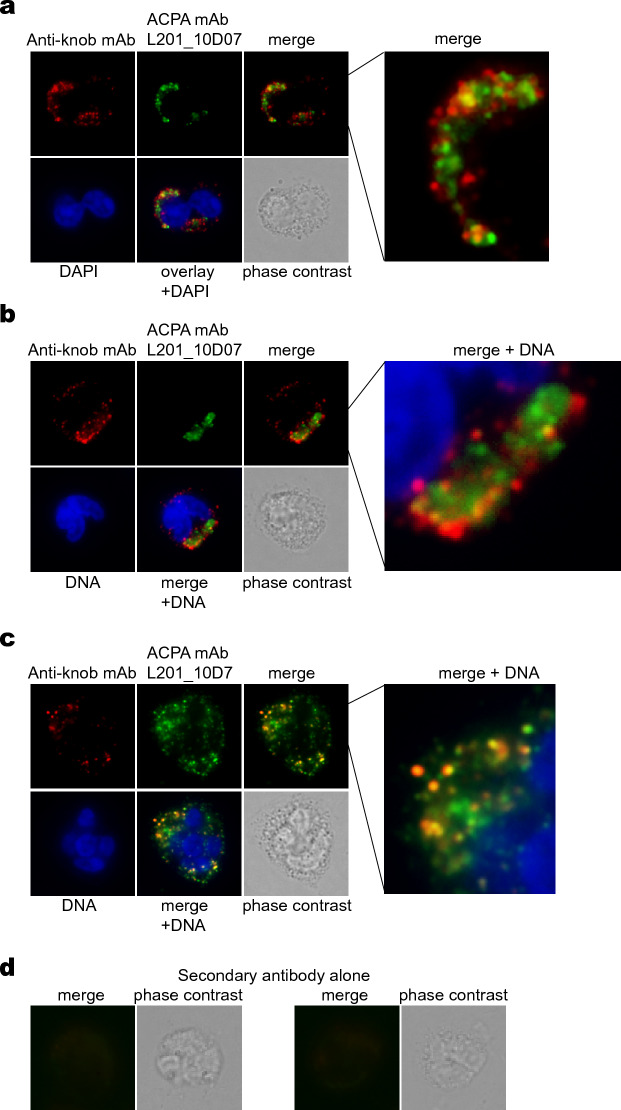
Citrullination in freshly isolated RA neutrophils with poly-perforin pores. (**a**) A representative neutrophil from an RA patient stained with the anti-knob mAb and the ACPA mAb L201_10D07. (**b**) Another neutrophil from a second RA patient. (**c**) A third representative neutrophil from a third RA patient. (**d**) Control staining of two RA neutrophils with secondary antibody alone.

When RA neutrophils were stained with the anti-knob mAb and L201_10D07 mAb together, we observed that many neutrophils were positive for both (Fig. 3a–c). The staining was closely adjacent but only partly overlapping. For example, in those neutrophils with perforin pores concentrated in one end of the cell, the increased reactivity with L201_10D07 was in the same end (Fig. 3a,b). Neutrophils with more dispersed perforin pores also had more widespread citrullination (Fig. 3c). Staining with the fluorophore-conjugated secondary antibodies alone did not result in any staining at all (Fig. 3d). Neutrophils with poly-perforin pores and intracellular citrullination from another RA patient are shown in Supplemental Fig. S4.

### Consequences of perforin-mediated intracellular citrullination in neutrophils

**Figure 4:**
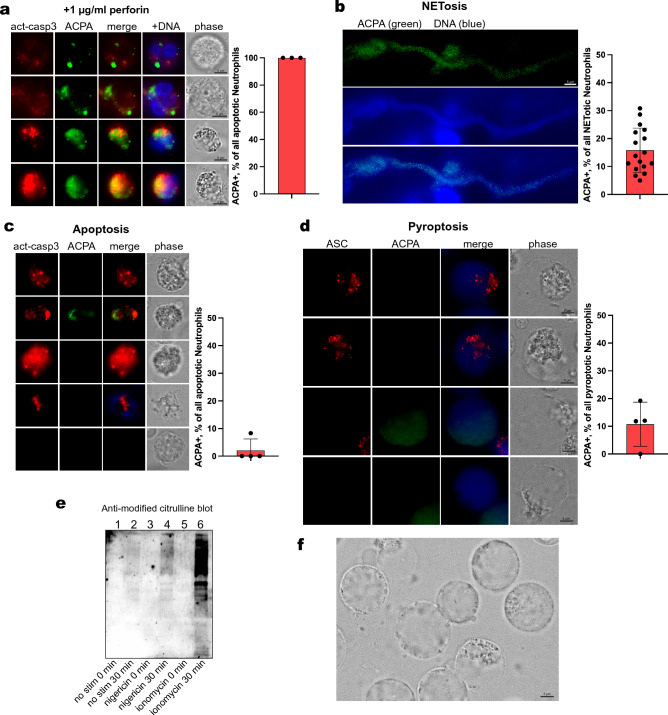
Citrullination during programmed cell death. (**a**) A neutrophil treated with perforin with activated caspase 3 and citrullination. (**b**) Neutrophils in NETosis after treatment with 100 nM phorbol ester. (**c**) Four neutrophils undergoing apoptosis after treatment with 1 µM staurosporine and stained for activated caspase 3 and citrullination. The last neutrophil (bottom row) is not in apoptosis. (**d**) Neutrophils in various stages of pyroptosis after treatment with 10 µM nigericin and stained with anti-ASC and ACPA mAb L201_10D07. Note the morphology of the cells and the faint ACPA staining in the third cell. (**e**) anti-modified citrulline immunoblot of lysates from neutrophils treated as indicated. (**f**) neutrophils in late-stage pyroptosis.

During target cell killing by cytotoxic T cells or natural killer (NK) cells, monomeric perforin is secreted onto the target cell plasma membrane, where it polymerizes into pores consisting of 20–24 monomers, as illustrated schematically in Fig. 1a. These pores allow for a rapid influx of Ca^2+^ down its 10,000-fold concentration gradient from the extracellular 2.2–2.6 mM to intracellularly around 150 nM. To induce effective target cell death by apoptosis, the cytotoxic lymphocyte also secretes granzyme B, a protease that activates the caspase cascade leading to apoptosis. For these reasons, we had expected that the freshly isolated RA neutrophils with perforin pores would rapidly enter apoptosis. However, following the neutrophils on cultures for up to 4 h revealed very few neutrophils undergoing apoptosis, visualized by staining for activated caspase-3. In contrast, cultures treated with 1 µg/ml of perforin for 4 h contained a number of neutrophils with activated caspase-3, every single one of which also stained positive for intracellular citrullination (Fig. 4a). Neutrophils with intracellular citrullination, but not in apoptosis, were also present. Furthermore, cultures of RA neutrophils with or without added perforin viewed after 18 h at 37 °C contained a mixture of viable cells and cells at various stages of programmed cell death via apoptosis, pyroptosis, or NETosis, but with no discernible differences in ratios of death pathways between samples containing only endogenous perforin pores or those with added perforin. Hence, it is clear that freshly isolated RA neutrophils with poly-perforin pores are not doomed to die quickly but can remain viable in cell culture as long as neutrophils without such pores.

### Citrullination during apoptosis, pyroptosis, and NETosis

It has been suggested that neutrophil extrusion of NETs, a process referred to as NETosis, is accompanied by clinically relevant protein citrullination in RA^10^. To compare this, and other forms of cell death, with the poly-perforin mediated leukotoxic hypercitrullination, we treated neutrophils with agents to induce NETosis (100 nM phorbol ester), apoptosis (1 µM staurosporine), or pyroptosis (10 µM nigericin). Apoptosis, pyroptosis and NETosis were discerned based on morphological features: of the neutrophils undergoing NETosis, as indicated by a greatly enlarged DNA staining often reaching extended shapes over 100 µm in length (Fig. 4b), 85% remained entirely negative for citrullination, while 1 in 6 showed a finely granular staining over much of the surface area of DNA (Fig. 4b and Supplemental Fig. S5). An identical pattern was seen with an antibody specific for citrullinated histone H3 (not shown). Apoptotic cells, identified by an antibody against active caspase-3 (Fig. 4c), were somewhat reduced in size, had a more irregular shape, and rapidly lost DNA staining, but were uniformly negative for citrullination (Fig. 4c) except in one single instance (second row in Fig. 4c). Pyroptosis, on the other hand, was readily recognized as a swelling of the neutrophils starting as a partial membrane enlargement and progressing to a balloon-like cell nearly twice in diameter and with a displaced intracellular aggregate (Fig. 4d,f). These cells contained aggregated inflammasomes (stained for ASC) but remained negative for citrullination until late-stage disruption of the membrane, at which time a faint and diffuse citrullination signal was observed (Fig. 4f). By anti-modified citrulline immunoblotting, pyroptotic neutrophils contained barely discernible citrullination compared to ionomycin-treated neutrophils (Fig. 4e). We conclude that perforin-induced citrullination is much more intense and consistent than citrullination induced by other pathways of programmed cell death, as was previously reported for apoptosis^22^.

### Neutrophil killing in Felty’s syndrome is accompanied by exceptionally high ACPA titers

**Figure 5:**
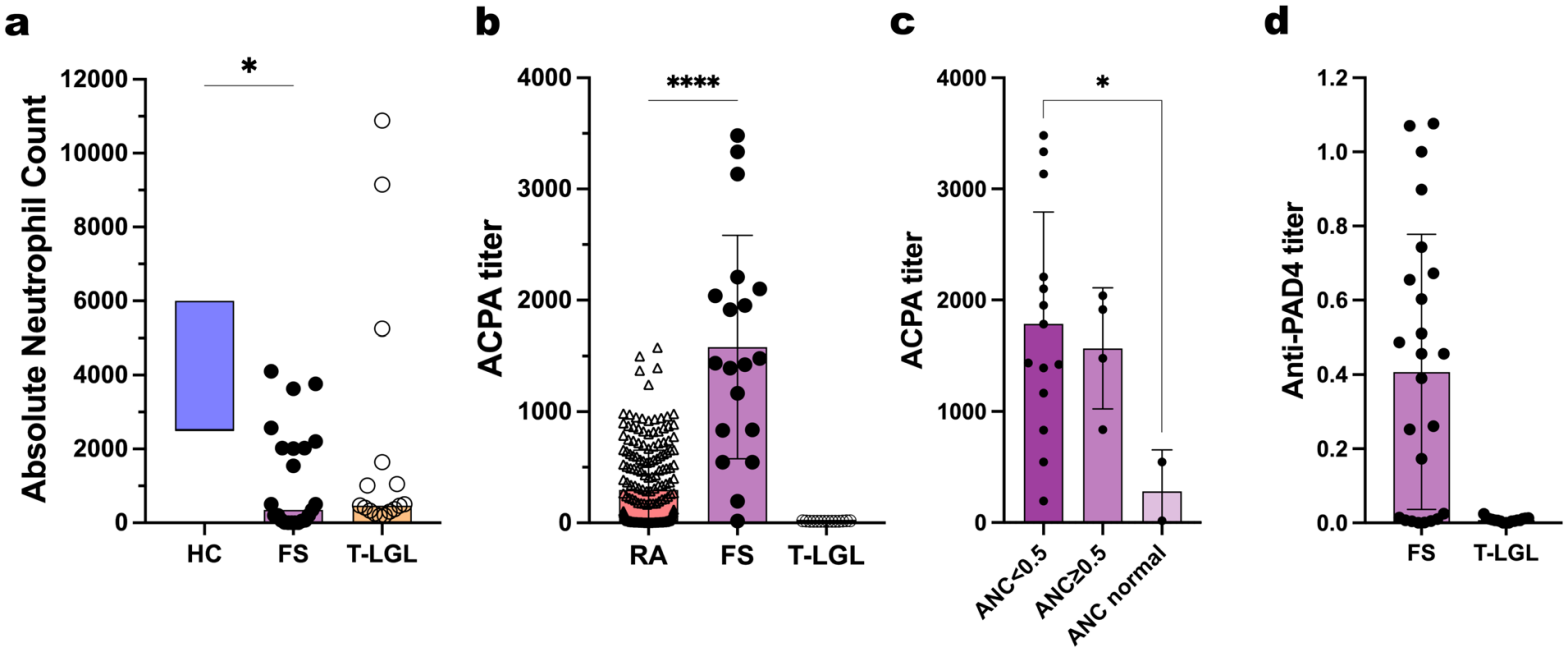
Neutropenia and high ACPA in Felty’s syndrome. (**a**) Absolute neutrophil counts (ANC) in patients with FS or T-LGL, compared to the normal range of ANC (HC, blue). (**b**) ACPA titers in patients with RA (n = 203), FS (n = 19), or T-LGL (n = 15). (**c**) ACPA titers in FS patients with severe or mild neutropenia or ANC within the normal range. (**d**) Anti-PAD4 autoantibody titers in patients with FS and T-LGL.

In further support of the notion that neutrophil targeting by perforin results in clinically relevant protein citrullination, we turned to a cohort of patients with Felty’s syndrome (FS; n = 20). This syndrome is an aggressive form of RA, in which expanded and activated cytotoxic lymphocytes accompany a severe neutropenia (Fig. 5a). T-cell Large Granular Lymphocyte Leukemia (T-LGL) is a very similar disease, except that the expanded cytotoxic lymphocytes are malignant. Approximately half of T-LGL patients develop RA^23,24^. As shown in Fig. 5b, ACPA titers were extraordinarily high in the FS patients compared to RA (n = 203), while patients with T-LGL without arthritis (n = 14) had none. FS patients with severe neutropenia (absolute neutrophil count < 0.5 × 10^9^/l) had higher ACPA titers than those with modest neutropenia (ANC ≥ 0.5, but below normal range), and particularly those in the normal range (2.5–4 × 10^9^/l) (Fig. 5c). Furthermore, many of the FS patients had autoantibodies against PAD4, while none of the T-LGL did (Fig. 5d). These data indicate that neutrophil killing by cytotoxic lymphocytes is accompanied by exceptionally prominent anti-citrulline immunity and aggressive RA. The presence of autoantibodies against PAD4 in FS also supports the involvement of this enzyme^25–30^.

## Discussion

Our findings are compatible with the notion that cytotoxic lymphocytes^31^ target neutrophils in RA by secreting perforin that creates poly-perforin pores in their plasma membrane that allow a rapid influx of calcium, which, in turn, leads to extensive intracellular citrullination by PAD4 (and perhaps PAD2). We propose that this process is sufficiently modest in seropositive RA that it does not result in noticeable neutropenia as it does not result in a rapid demise of the targeted neutrophils and perhaps is also counterbalanced by compensatory neutropoiesis. In Felty’s syndrome, on the other hand, neutrophil killing in the bone marrow^32^ outpaces compensatory neutropoiesis and therefore results in severe neutropenia as well as in excessive citrullination and high titers of ACPA (Fig. 5b). In contrast, in RA the neutrophils in circulation that have intensely staining dots of perforin include many with a mature multi-segmented nuclear morphology. If these were exposed to cytotoxic lymphocytes in the bone marrow, they must have survived the encounter for several days. More likely, they were hit after leaving the bone marrow. Nevertheless, our findings introduce the possibility that perforin pores and intracellular citrullination may not always kill. A sublethal perforin targeting would be reminiscent of the ability of macrophages to survive gasderminD polymerization during pyroptosis by a rapid repair process to restore plasma membrane integrity. Perhaps neutrophils can repair their membrane after perforin pore formation to restore its integrity and allow them to remain viable. It is also possible that a subset of neutrophils acquires too many perforin pores to survive in the RA patient and therefore vanish from circulation before we can observe them. If this is the case, the upper limit of 5 or 6 pores per neutrophil that we detect (Fig. 2f) may represent a threshold number of pores a neutrophil can tolerate.

Further work will be needed to explain why cytotoxic lymphocytes target neutrophils in RA. We assume that they recognize an (auto)antigen on the surface of neutrophils, triggering them to target the neutrophil through an immunological synapse to deliver a bolus of perforin from their secretory granules. Since many cytotoxic T cells in RA contain granzyme K^33^, rather than granzyme B, this may not effectively induce apoptosis in the targeted neutrophils, explaining why they seem to remain viable.

Our study has some caveats. We cannot categorically exclude the possibility that perforin pore formation on neutrophils occurred after venipuncture or during neutrophil isolation. Its complete absence in healthy donors argues against this possibility and we are not aware of precedents for cytotoxic lymphocyte action at reduced temperature in ex vivo blood samples. We would also expect such targeting to result in apoptosis shortly after isolation, which we do not observe. A second caveat is regarding the causality of neutrophil citrullination for the immune response that results in ACPA. The exceptionally high ACPA titers in FS patients with the most severe neutropenia and the fact that of some of the citrullinated proteins we detect in perforin-treated neutrophils are present in their citrullinated form in the synovial fluid from RA patients^34^ support a connection between intracellular neutrophil citrullination, ACPA formation, and RA disease activity. A true causality can only be established through clinical trials with PAD inhibitors or agents that interfere with neutrophil targeting by perforin. Nevertheless, our study is the first to demonstrate that neutrophil targeting by perforin occurs in RA patients.

## Online methods

### RA Patient cohort

A cohort of patients with RA (n = 30) and healthy individuals (n = 20) were recruited through the University of Washington, Division of Rheumatology Biorepository to participate in research studies at the University of Washington Medical Center, Seattle, WA. Patients with RA met the classification criteria for RA established by the American College of Rheumatology (ACR) and the European League Against Rheumatism (EULAR) in 2010. Healthy donors were obtained through the exact same procedure. Institutional Review Board approval for our study was obtained from the University of Washington ethics board (STUDY00006196) and informed written consent was obtained from all participants. All experiments were performed in accordance with relevant guidelines and regulations.

### Felty’s syndrome and large granular T cell leukemia patients

Serum samples from consented T-LGL leukemia patients with RA (n = 20; FS) or without co-existing RA (n = 17) were from the LGL Leukemia Registry at the University of Virginia, Charlottesville, VA under IRB-approved protocols (IRB-HSR#1700 and IRB #17070) by Dr. Thomas P. Loughran Jr. Information on patient absolute neutrophil count (ANC) at blood draw, and current and past use of glucocorticosteroid, biologic and non-biologic disease modifying anti-rheumatic drugs (DMARDs) and/or use of anti-granulocyte stimulating factor as well as use of any chemotherapy obtained for T-LGL leukemia was queried from the Registry and was available for all patients with the exception of ANC count for one T-LGL/RA patient.

### ACPA testing by CCP assay

Serum from patients or healthy controls were analyzed ACPA by CCPlus (Immunoscan, Euro Diagnostica, Sweden) according to the manufacturer’s instructions.

### Perforin ELISA

3.3 µg/ml of purified perforin-1 protein (Enzo Life Sciences # 50-225-2676) was adsorbed onto 96-well polystyrene plates in 0.1 M bicarbonate (pH 9.6) buffer overnight, washed in phosphate-buffered saline with 0.05% Tween, and blocked in 2% bovine serum albumin (BSA) in phosphate-buffered saline for 2 h. Plasma was added at 0.5% in blocking buffer for overnight incubation at 4 °C, washed extensively, and then incubated with 1:2,000 dilution of horse radish peroxidase-conjugated anti-human IgG. The reaction was then washed, and developed with TMB, with the color reaction terminated with 2N sulfuric acid, and the absorbance measured at 450 nm using a plate reader.

### Production of anti-knob mAb

Based on the structure of poly-perforin pores determined by the X-ray crystallography and cryo-electron microscopy^35^, we selected the peptide that constitutes most of the N-terminal knob (amino acid residues 24–46, HTAARSECKRSHKFVPGAWLAGE) and coupled it covalently to keyhole limpet hemocyanin for immunization of mice by Ameritek Inc. (Everett, WA). The animals were boosted with the same peptide covalently coupled to bovine serum albumin, which was also used for initial screening. The resulting hybridoma 4G10 was adapted to serum-free medium and the mAb purified by ion exchange chromatography. The mAb was validated by ELISA against purified human perforin, which gave it an approximate EC_50_ of 0.1 nM, and by immunofluorescence staining of cells treated with purified perforin, as shown in Fig. 1.

### Other antibodies

The L201_10D07 mAb was derived from a lung memory B cell from an ACPA-positive RA risk individual and subsequently expressed as recombinant human IgG1 antibodies, as previously described^21^. It immunoprecipitates several citrullinated proteins (*e.g*., actin, α-enolase, filamin, myosin-9, myosin light chain 6, PAD4, plastin-2, and vimentin), but it remains unclear which proteins it recognizes directly. Anti-activated caspase-3 ab32042 (Abcam, #E83-77). Anti-ASC mAb sc-514414 (Santa Cruz). Alexa Fluor conjugated goat anti-mouse IgG Ab AF647 (Invitrogen, #A-21235), goat anti-mouse IgG (H + L) Ab AF488, (Invitrogen, #A-11001), goat anti-human IgG Ab AF555 (Invitrogen, #A21433), goat anti-Rabbit IgG Ab AF647 (Invitrogen, #A-21245).

### Neutrophil isolation from RA patients and healthy controls

Neutrophils were isolated from venous blood samples by PolymorphPrep separation (CosmoBio, USA) according to the manufacturer's instruction. Neutrophils were cultured in DMEM high glucose (Thermo Fisher Scientific #11-965-118) on a glass-bottom 96-well plate (Cellvis, #P96-1-N) pre-coated with poly-L-lysine (Millipore-Sigma # P4707) for 30 min at 37 °C to allow the cells to adhere. The cells were then treated without or with 1 µg/ml Perforin (Enzo Life Sciences, USA), 2 µM Ionomycin A23187 (Millipore-Sigma, Germany, #10634), 10 µM nigericin, or 100 nM phorbol myristate (Invitrogen,) at 37 °C for various times.

### Immunofluorescence microscopy

For immunofluorescence microscopy, neutrophils were fixed with 4% paraformaldehyde solution (ThermoFisher Scientific, USA, #AAJ19943K2) for 20 min at room temperature, followed by two washes with phosphate-buffered saline (PBS). Cells were permeabilized using 0.25% Triton X-100 (Millipore-Sigma, Germany, #9036-19-5) in PBS for 10 min at room temperature followed by three washes with PBS. To prevent nonspecific binding, neutrophils were blocked using 10% normal goat serum (ThermoFisher, #50062Z) in BlockAid solution (ThermoFisher Scientific, #B10710) and human Fc gamma RII/CD32 antibody (Fisher Scientific, USA, # MAB1300SP) overnight at 4 °C. Neutrophils were then incubated with a 1:50 dilution of mouse anti-perforin antibody (Ameritek, Everett, WA, USA) for 1 h at RT, followed by three washes with PBS.

Next, the cells were stained with goat anti-mouse IgG secondary Ab labelled with either Alexa Fluor 488 or Alexa Fluor 647 (ThermoFisher Scientific, USA) and Alexa Fluor 488-labeled monoclonal ACPAs (obtained from Karolinska Institute, Stockholm, Sweden) for 45 min at RT. Nuclei were stained with either DAPI solution (ThermoFisher Scientific, USA, #62248) or Hoechst33342 solution (ThermoFisher Scientific, USA, # PI62249). Images were captured using a Keyence BZ-X800 Fluorescence microscope and analyzed using BZ-X800 Analyzer software.

### Mass spectrometry

For LC–MS/MS analysis, 10^6^ neutrophils were digested with trypsin and the resulting peptides subjected to liquid chromatography (LC)-tandem mass spectrometry (MS/MS) in an Orbitrap Fusion Lumos Tribrid instrument. Mass spectra were filtered to only include proteins with a probability of match = 1 and ranked by the precision test for fragment match with a value of q < 10^–4^.

### Gel electrophoresis and anti-modified citrulline immunoblotting

Neutrophils were lysed by mixing 10^7^ cells in 500 µl lysis buffer with an equal volume of twice concentrated SDS sample buffer, heated at 95 °C, and clarified by centrifugation. 35–45 µl (0.35–0.45 × 10^6^ cell equivalents) samples were resolved by SDS gel electrophoresis and transferred to polyvinylidene fluoride membranes, which were chemically modified as per the manufacturer’s protocol, blocked with Superblock and detected with anti-modified citrulline antibody (MilliporeSigma) at 1:4000, 4 °C, overnight), horse-radish-conjugated anti-mouse Ab and developed by enhanced chemiluminescence detection.

### Supplementary Information

Supplementary Information 1.

## Data Availability

The datasets used and/or analyzed during the current study are available from the corresponding author on reasonable request.
