# Supplementary material for: Evidence of membranolytic targeting and intracellular citrullination in neutrophils isolated from patients with rheumatoid arthritis
Source: Sci Rep. 2024 Jul 5;14:15511. doi: 10.1038/s41598-024-66516-w (PMC11226660; doi:10.1038/s41598-024-66516-w)

**SUPPLEMENTAL Material**

**Table S1. Proteins citrullinated after addition of 1 µg/ml perforin.**

**Fig. S1 | RA monocytes with poly-perforin pores.** Fifteen representative monocytes with positive anti-knob mAb staining at 100X magnification.

**Fig. S2 |** **ACPA mAb L201_10D07 detects intracellular citrullination after treatment of HC neutrophils with Ionomycin.** **Upper left panels**, Four representative neutrophils without treatment. **Upper right panels**, Four representative neutrophils after treatment with 1 µM ionomycin for 30 min at 37°C. **Lower left panels,** uncropped 40X view of untreated neutrophils. **Lower right panels,** uncropped 40X view of neutrophils after treatment with 1 µM ionomycin for 30 min at 37°C.

**Fig. S3 | A subset of RA, but not HC, neutrophils are reactive with ACPA mAb L201_10D07 without any treatment.** **Top panel**, uncropped 40X view of untreated RA neutrophils. **Middle panel**, uncropped 60X view of untreated RA neutrophils. **Bottom panel,** uncropped 40X view of untreated HC neutrophils.

**Fig. S4 |** **Poly-perforin pores on freshly isolated untreated RA, but not HC, neutrophils.**  **Top panel**, uncropped 100X view of untreated RA neutrophils. **Bottom panel,** uncropped 100X view of untreated HC neutrophils

**Fig. S5 | RA neutrophils undergoing** **NETosis and stained with ACPA mAb L201_10D07.** Five representative neutrophils with positive ACPA staining at 100X magnification. Bottom panel, a representative uncropped image showing multiple NETotic neutrophils, of which only one is positive for citrullination.

**Fig. S6 | Panel e from Fig. 4 as uncut gel**

**Supplemental Table S1. Proteins citrullinated after addition of 1 µg/ml perforin**

Gene (protein) q value peptide sequence

ACTA, ACTS, ACTG, ACTB, ACTC (actin) 0.000572 R.LDLAGR[cit]DLTDYLMK

ACTN4 (α-actinin 4) 0.000591 K.MLDAEDIVNTAR[cit]PDEK.A

AZU1 (azurocidin) 5.68E-20 R.R[cit]GGICNGDGGTPLVCEGLAHGVASFSLGPCGR.G

COROA1 (coronin 1A) 3.50E-10 R.R[cit]AAPEASGTPSSDAVSR.L

CP4FC (cytochrome P450) 0.000503 R.R[cit]TLPTQGIDDFFK.D

CTSG (cathepsin G) 1.38E-06 R.AQEGLR[cit]PGTLCTVAGWGR.V

H1.5 (histone 1.5) 0.00286 K.ER[cit]NGLSLAALK.K

H2A1 (histone H2A type 1) 1.19E-05 R.SSR[cit]AGLQFPVGR.V

HNRNPU (heterogeneous nuclear ribonucleoprotein U) 1.02E-10 R.LQAALDDEEAGGR[cit]PAMEPGNGSLDLGGDSAGR.S

HP1BP3 (heterochromatin protein 1-binding protein 3) 9.93E-06 K.NR[cit]SSAVDPEPQVK.L

LRCH4 (leucine-rich repeat and calponin homology domain-containing protein 4) 0.00555 R.GSALGDLAPSR[cit]PPSFSPCPAEDLFPGHR.Y

MMP9 (matrix metalloproteinase 9) 2.52E-06 R.VAEMR[cit]GESK.S

MRM3 (rRNA methyltransferase 3, mitochondrial) 0.0075 K.APSEASAQEQR[cit]EK.Q

PHF3 (PHD finger protein 3) 0.000166 R.LSHGDR[cit]GTDGK.A

POTEE (POTE ankyrin domain family member E) 5.98E-05 K.QEYDESGPSIVHR[cit]K.C

PRF1 (perforin-1) 9.24E-07 R.R[cit]SGSFPVDTQR.F

PSB8 (proteasome subunit beta type-8) 3.39E-08 R.LSGNMFSTGSGNTYAYGVMDSGYR[cit]PNLSPEEAYDLGR.R

RIPK4 (receptor-interacting protein kinase 4) 0.00822 K.SR[cit]LFDTK.H

SNRPD2 (small nuclear ribonucleoprotein Sm D2) 0.00014 K.R[cit]EEEEFNTGPLSVLTQSVK.N

TKT (transketolase) 5.99E-05 K.GHAAPILYAVWAEAGFLAEAELLNLR[cit]K.L

TRFL (lactotransferrin) 1.76E-06 K.FGR[cit]NGSDCPDK.F

VIME (vimentin) 2.67E-05 K.SR[cit]LGDLYEEEMR.E

ZN574 (zinc finger protein 574) 0.00909 R.EHR[cit]CAAAAAQAPR.R

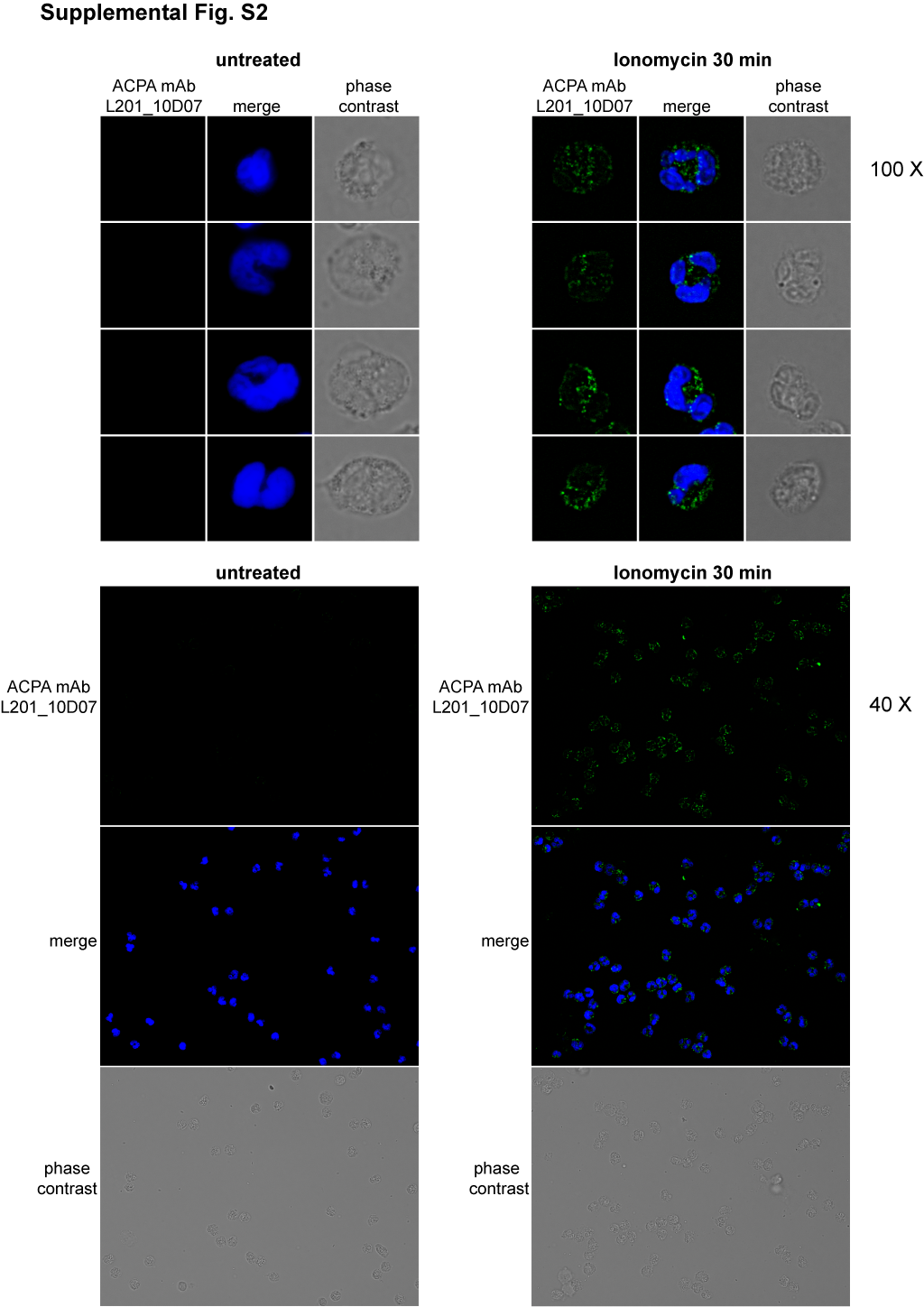


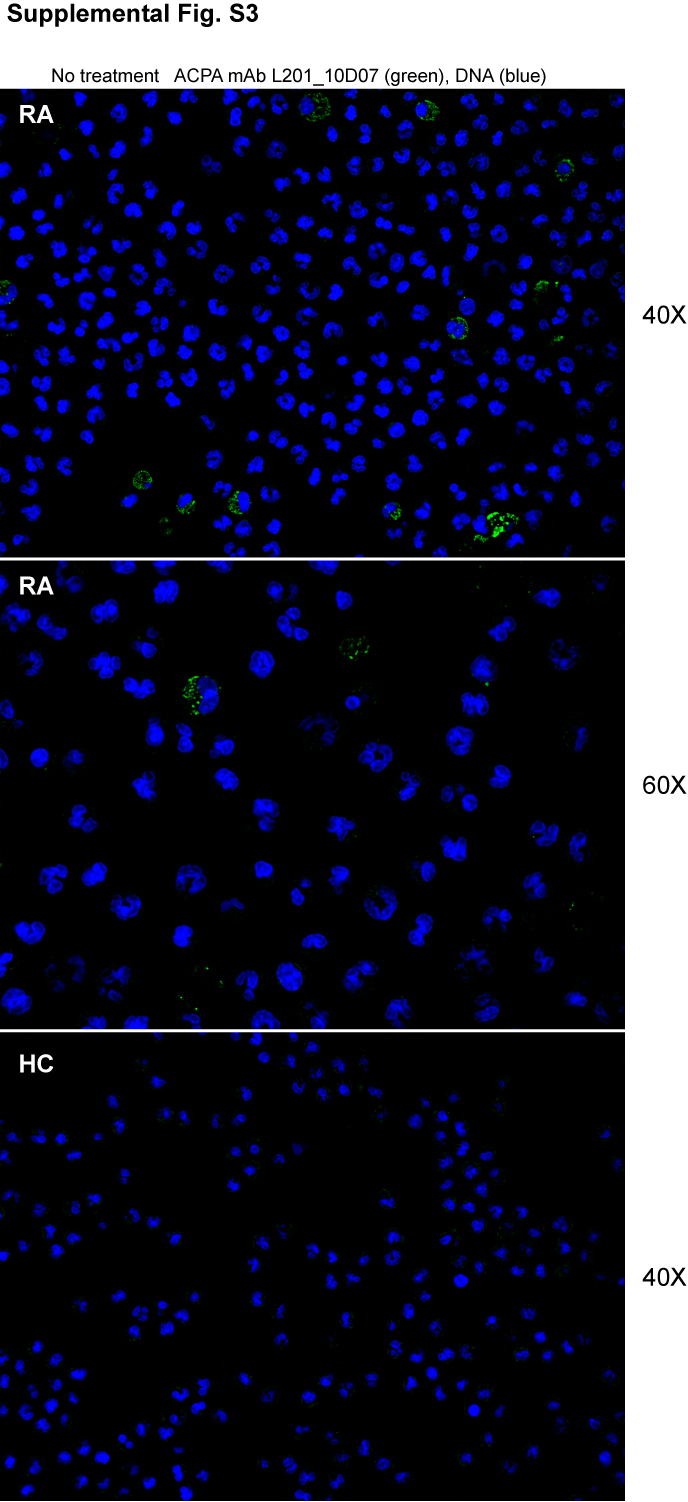


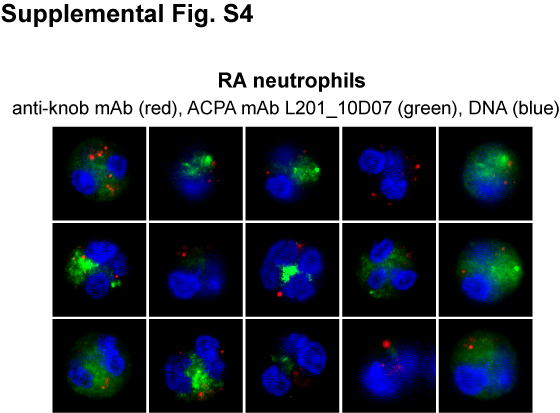


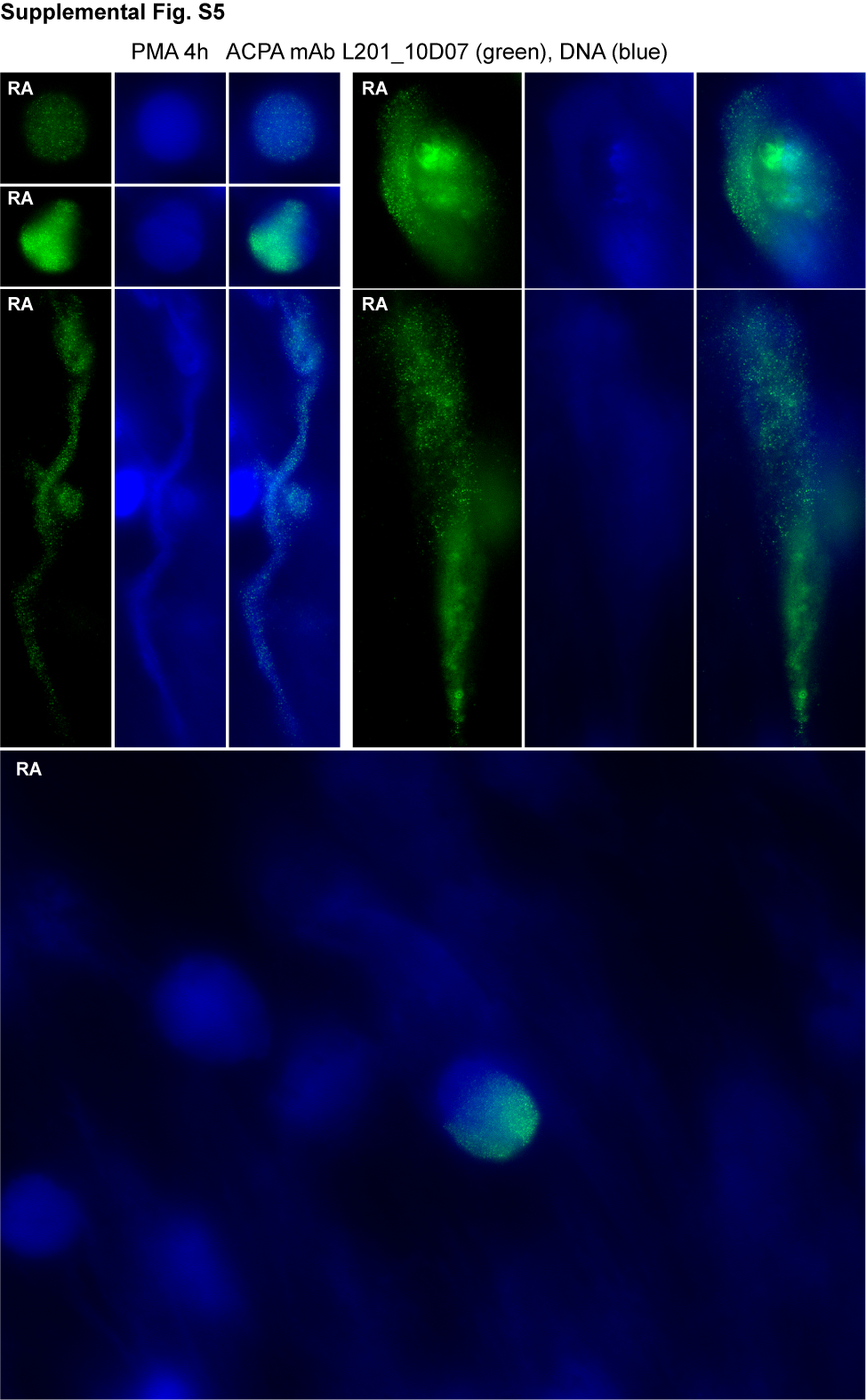


**Supplemental Fig. S6**


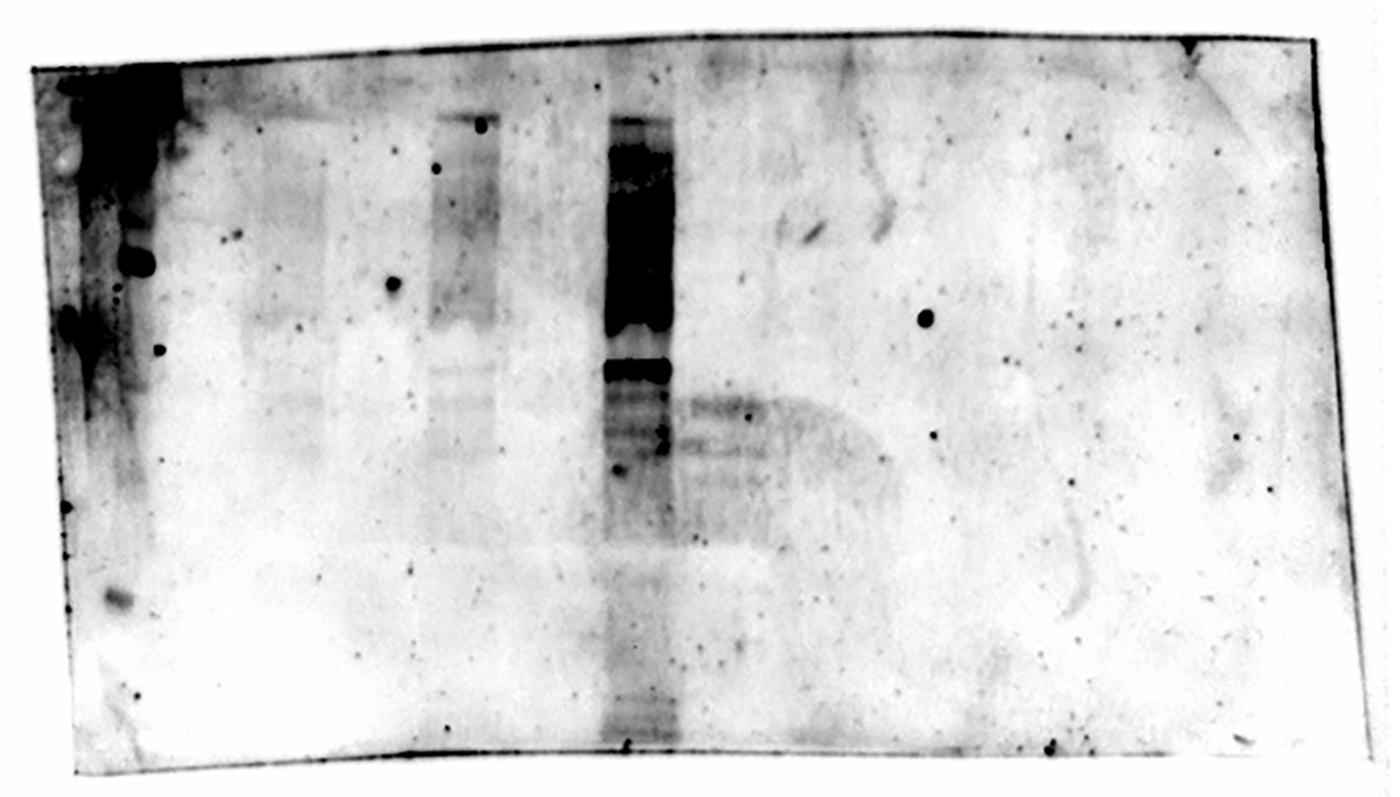

Supplement: Supplementary file 1 — Supplementary Information 1. [file 41598_2024_66516_MOESM1_ESM.docx]
